# Overexpression of endothelial nitric oxide synthase suppresses features of allergic asthma in mice

**DOI:** 10.1186/1465-9921-7-58

**Published:** 2006-04-05

**Authors:** Robert Ten Broeke, Rini De Crom, Rien Van Haperen, Vivienne Verweij, Thea Leusink-Muis, Ingrid Van Ark, Fred De Clerck, Frans P Nijkamp, Gert Folkerts

**Affiliations:** 1 Department of Pharmacology and Pathophysiology, Utrecht Institute for Pharmaceutical Sciences, Utrecht University, P.O. Box 80.082, 3508 TB Utrecht, The Netherlands; 2 St Antonius Hospital, Nieuwegein, The Netherlands; 3 Department of Cell Biology & Genetics, Erasmus Medical Centre, Rotterdam, The Netherlands; 4 Department of Vascular Surgery, Erasmus Medical Centre, Rotterdam, The Netherlands; 5 Janssen Research Foundation, Beerse, Belgium

## Abstract

**Background:**

Asthma is associated with airway hyperresponsiveness and enhanced T-cell number/activity on one hand and increased levels of exhaled nitric oxide (NO) with expression of inducible NO synthase (iNOS) on the other hand. These findings are in paradox, as NO also relaxes airway smooth muscle and has immunosuppressive properties. The exact role of the endothelial NOS (eNOS) isoform in asthma is still unknown. We hypothezised that a delicate regulation in the production of NO and its bioactive forms by eNOS might be the key to the pathogenesis of asthma.

**Methods:**

The contribution of eNOS on the development of asthmatic features was examined. We used transgenic mice that overexpress eNOS and measured characteristic features of allergic asthma after sensitisation and challenge of these mice with the allergen ovalbumin.

**Results:**

eNOS overexpression resulted in both increased eNOS activity and NO production in the lungs. Isolated thoracic lymph nodes cells from eNOS overexpressing mice that have been sensitized and challenged with ovalbumin produced significantly less of the cytokines IFN-γ, IL-5 and IL-10. No difference in serum IgE levels could be found. Further, there was a 50% reduction in the number of lymphocytes and eosinophils in the lung lavage fluid of these animals. Finally, airway hyperresponsiveness to methacholine was abolished in eNOS overexpressing mice.

**Conclusion:**

These findings demonstrate that eNOS overexpression attenuates both airway inflammation and airway hyperresponsiveness in a model of allergic asthma. We suggest that a delicate balance in the production of bioactive forms of NO derived from eNOS might be essential in the pathophysiology of asthma.

## Background

Asthma is a chronic inflammatory disease of the airways characterized by airway obstruction, epithelial damage and airway hyperresponsiveness [1,2]. The increased airway responsiveness is believed to be the result of airway inflammation as well as epithelial damage [3-5]. There is increasing evidence that activated T lymphocytes modulate the pathogenesis of asthma [6,7]. Specifically, increased numbers of CD4^+ ^T cells (Th2) have been found in the bronchial mucosa of asthmatic patients, with the consequent elevated levels of interleukin-5 (IL-5) and IL-10 [8-10]. Moreover, interferon-γ (IFN-γ) secreting T cells (Th1) were increased in bronchoalveolar lavage (BAL) fluid of asthmatic patients [11,12] and it has been reported that these T cells can induce airway inflammation with neutrophilic inflammation [13,14]. Therefore, both Th1 and Th2 cells are important in airway inflammation and asthma [15]. In addition, inflammatory cells like eosinophils, macrophages and neutrophils are capable of releasing cytokines, proteases, reactive oxygen species and lipid mediators that contribute to the pathogenesis of asthma and the development of airway hyperresponsiveness [3-5,16-18].

Nitric oxide (NO) regulates many physiological processes [19]. NO itself has a short half-life (1–5 s) because of its reactivity with various biological compounds. On one hand NO might react with reactive oxygen species resulting in nitrosative stress; on the other hand it might modificate cysteine sulphurs to form S-nitrosothiols [20], of which S-nitrosoglutathione represents a major source of bronchodilatory NO activity [21] NO is synthesized from L-arginine by the enzyme NO synthase (NOS), which exists in three distinct isoforms: constitutive neural NOS (nNOS), inducible NOS (iNOS) and constitutive endothelial or epithelial NOS (eNOS). The isoforms are products of distinct genes located on different human chromosomes, each with a characteristic pattern of tissue-specific expression. NO produced by cNOS acts as a signalling molecule in several processes, including regulation of vascular and airway smooth muscle tone [19,22]. The role of this calcium-dependent isoform in the airways has been previously investigated. Incubation of NOS inhibitors on the mucosal side of the guinea pig trachea induced an increased contractile response to histamine and cholinergic agonists [23]. Moreover, a NO deficiency in the airways contributes to the development of airway hyperresponsiveness in animal models for asthma, i.e. guinea pigs with a viral respiratory tract infection [24] and ovalbumin-sensitized and challenged guinea pigs [25,26]. Inhalation of exogenous NO causes bronchodilation in asthmatic patients [27] and inhibition of NO production by NO synthase inhibitors increases airway responsiveness in patients with mild asthma [28,29]. The calcium-independent iNOS is induced in a wide variety of cell types by several cytokines. NO production by iNOS is several times higher than by cNOS [30]. In the airway epithelium of human asthmatics, increased expression of iNOS has been found [30,31] and high levels of NO produced by this NOS isoform is thought to reflect the increased exhaled NO levels found in asthmatic patients [32,33]. Therefore, it has been suggested that NO produced by iNOS is a marker of inflammation.

Several studies have shown the importance of the different NOS isoforms in asthma, either by using selective NOS inhibitors or by using mouse strains with deletions in one of the NOS genes. However, no studies have been performed to investigate the effects of overexpression of one of the NOS isoforms on the development of asthmatic features. Furthermore, although the importance of iNOS in asthma is well established [30,34], the precise role of eNOS is still unknown. Interestingly, recent studies suggest that eNOS gene polymorphisms are implicated in asthma [35-38]. In the presence of epithelial dysfunction, these polymorphisms may have clinical significance [39].

In the present study, we examined the contribution of eNOS on the development of asthmatic features. We used transgenic mice that overexpress eNOS and measured characteristic features of allergic asthma after sensitisation and challenge of these mice with the allergen ovalbumin. We found that eNOS overexpression prevents the development of airway hyperresponsiveness. Furthermore, there was a reduction in the number of inflammatory cells, especially eosinophils, in the lungs and an attenuated production of cytokines by lymph node cells in these animals. We conclude that eNOS overexpression result in attenuation of both airway hyperresponsiveness and airway inflammation.

## Methods

### Generation of eNOS transgenic mice

A genomic clone has been isolated from a home-made cosmid library [40]. It comprises the complete, unmodified gene encoding endothelial NO synthase (eNOS, NOS3) plus its natural flanking sequences. These consist of ~ 6 kb of the 5' sequence, including the natural promotor, and ~ 3 kb of the 3' sequence. Vector sequences were removed by restriction endonuclease digestion and DNA was dissolved in micro-injection buffer (10 mmol/L Tris-HCl, pH 7.5; 0.1 mmol/L EDTA) at a concentration of 2 μg/ml. The DNA was micro-injected into fertilized oocytes from FVB mice. These oocytes were transferred into the oviducts of pseudopregnant foster females. Mice used in the present study were backcrossed for at least 5 generations onto a C57BL/6 background (>96% C57BL/6), the controls (WT) for the eNOS overexpressing mice (eNOS). The mice were housed in macrolon cages and provided with food and water ad libitum. All animal experiments were performed in compliance with the Guidelines of the Ethical Committee on the Use of Laboratory Animals of The University Utrecht and Erasmus University Rotterdam.

### eNOS activity measurements

eNOS activity was measured in lung tissue by the L-arginine to L-citrulline conversion assay using a nitric oxide synthase assay kit (Calbiochem, La Jolla, CA, USA; cat. no. 482700) according to the manufacturer's instructions.

### Western blot analysis of eNOS

Lung tissue isolated from control or eNOS overexpressing mice were homogenized in 1 ml of 50 mM Tris-HCl buffer, pH 7.8, containing 150 mM NaCl, 1 mM EDTA, 1 mM sodium vanadate, 1% NP-40, 1 mM phenylmethylsulfonyl fluoride (PMSF). The samples were transferred to eppendorf tubes and centrifuged at high speed in a microcentrifuge for 10 minutes at 4°C to remove insoluble material. Protein content was determined using the method of Bradford [41] with BSA as standard. Equal amounts of protein were added to a 0.1 M Tris buffer containing 50 μM dithiothreitol, 0.01% bromophenol blue, 1% sodium dodecyl sulfate (SDS) and 10% glycerol and boiled for 5 minutes. Proteins (30 μg/lane) were electrophoresed under reducing, denaturing conditions in 7.5% SDS polyacrylamide gel, transferred to nitrocellulose paper and probed with an antibody against humans eNOS (Santa Cruz Biotechnology, Inc., Santa Cruz, California, USA) or with an antibody against the phosphorylated site of eNOS (P-eNOS, Cell Signaling Technology, Beverly, MA, USA). Antibody binding was detected using an amplified alkaline phosphatase immuno blot kit (Biorad Laboratories, Hercules, California, USA) according to the manufacturer's recommendation.

### Sensitization and challenge

All mice were sensitized to ovalbumin (OVA; chicken egg albumin, grade V, Sigma, St. Louis, MO, USA). Active sensitization was performed by 2 intraperitoneal injections of 0.1 ml alum-precipitated antigen, comprising 10 μg OVA absorbed onto 2.25 mg alum (AlumImject; Pierce, Rockford, IL, USA) on day 0 and 14. Four weeks after the last injection, the mice were exposed either to an OVA (10 mg/ml in pyrogen-free saline, OVA group) or control solution (saline, SAL group) aerosol challenge for 20 minutes once daily on day 42, 45 and 48. The aerosol was performed in a plexiglass exposure chamber (5 L) coupled to a Pari LC Star nebulizer (PARI Respiratory Equipment, Richmond, VA, USA; particle size 2.5–3.1 μm) driven by compressed air at a flow rate of 6 L/min. Aerosol was given in groups composed of 6 animals.

### Measurement of airway responsiveness in vivo

Airway responsiveness was measured in conscious, unrestrained mice 24 hours after the last aerosol exposure using barometric whole-body plethysmography by recording respiratory pressure curves (Buxco; EMKA Technologies, Paris, France) in response to inhaled methacholine (acetyl-β-methyl-choline chloride, Sigma). Airway responsiveness was expressed in enhanced pause (Penh), which is a measure of bronchoconstriction, as described in detail previously [42]. Briefly, mice were placed in a whole-body chamber, and basal readings were obtained and averaged for 3 min. Aerosolized saline, followed by increasing doubling concentrations (1.56 – 25 mg/ml saline) of methacholine, were nebulized for 3 min, and readings were taken and averaged for 3 min after each nebulization.

### Analysis of cellular composition of bronchoalveolar lavage (BAL) fluid

Following airway responsiveness measurements, mice received a lethal dose of pentobarbitone sodium (Euthesate^® ^0.6 mg/kg body weight, intraperitoneally). The trachea was trimmed free of connective tissue and a small incision was made to insert a cannula in the trachea. Via this cannula, the lungs were filled with 1 ml aliquots of pyrogen free saline (37°C) supplemented with aprotinin (2 μg/ml, Sigma). Fluid was collected in plastic tubes on ice. This procedure was repeated 3 times with aliquots of pyrogen free saline and fluid was collected in a separate plastic tube on ice and cell suspensions recovered from each animal were pooled. BAL cells were centrifuged (400 × g, 4°C, 5 min) and supernatant from 1 ml aliquots were collected and stored (-30°C) until cytokines and NO were measured by ELISA and NO analyzer. The pellets from the first ml and 3 ml aliquots were pooled and resuspended in 150 μl PBS. The total number of cells in the BAL fluid was determined using a Bürker-Türk bright-line counting chamber (Karl Hecht Assistant KG, Sondheim/Rohm, Germany). For differential BAL fluid cell counts, cytospin preparations were made and stained with Diff-Quik (Dade AG, Düdingen, Switzerland). Per cytospin, 200 cells were counted and differentiated into alveolar macrophages, eosinophils, lymphocytes and neutrophils by light microscopical observation under oil immersion.

### Serum levels of total IgE

Blood samples were obtained from the mice via a cardiac puncture, left at room temperature for two hours and subsequently centrifuged for 10 min at 20,000 × g. Serum was collected and samples were kept at -20°C until analysis. Total IgE was measured using an ELISA method. Briefly, microtiter plates (Nunc A/S, Roskilde, Denmark) were coated overnight at 4°C with 2 μg/ml rat anti-mouse IgE (clone EM95) diluted in phosphate-buffered saline (PBS). The next day, the ELISA was performed at room temperature. After blocking with ELISA buffer (PBS containing 0.5% bovine serum albumin (Sigma), 2 mM EDTA, 136.9 mM NaCl, 50 mM Tris, 0.05% Tween-20 (Merck, Whitehouse Station, NJ, USA) pH 7.2) for 1 hour, serum samples diluted in ELISA buffer, were added for 2 hours. Thereafter, plates were incubated with 1 μg/ml second biotinylated antibody (Biotin anti-mouse IgE, PharMingen, San Diego, CA, USA) diluted in ELISA buffer for 1.5 hours. After washing, streptavidin-peroxidase (0.1 μg/ml, CLB, Amsterdam, The Netherlands) was added and incubation was performed for 1 hour. Color development was performed with o-phenylenediamine-dichloride substrate (0.4 mg/ml; Sigma) and 0.04% H_2_O_2 _in PBS and stopped by adding 4 M H_2_SO_4_. The optical density was read at 492 nm, using a Benchmark microplate reader (Bio-Rad Laboratories, Hercules, CA, USA).

### Determination of cytokine production by OVA-restimulated thoracic lymph nodes cells in vitro

Cytokine production by antigen-stimulated T cells derived from thoracic lymph nodes (TLN) was determined as described previously [43]. Briefly, TLN draining the lungs were removed, transferred to cold sterile PBS and filtered through a 70- μm nylon cell strainer (Becton Dickinson Labware, Franklin Lakes, NJ) with 10 ml RPMI 1640 to obtain a single-cell suspension. The lymph node cells were washed and resuspended in culture medium (RPMI 1640 containing 10% FCS, 1% glutamax I, and gentamicin (all from Life Technologies, Gaithersburg, MD, USA) and 50 mM -mercaptoethanol (Sigma). Cells were cultured in flat bottom 96-well plates (Greiner Bio-One GmbH, Kremsmuenster, Austria) at a concentration of 1 × 10^6 ^cells per ml in a volume of 200 μl. The cells were cultured for 5 days (37°C with 5% CO_2 _in humidified air) with culture medium or OVA (10 μg/ml). Each in vitro stimulation was performed in triplicate. After 5 days of culture, the supernatant was harvested, pooled per stimulation, and stored at -20°C until cytokine levels were determined by ELISA. The IFN-γ, IL-5 and IL-10 ELISAs were performed according to the manufacturer's instructions (PharMingen, San Diego, CA, USA).

### Determination of cytokine levels in BAL fluid

Cytokine levels were determined in supernatant of the first ml of the lavage fluid by ELISA (see above). IFN-γ and IL-10 levels in BAL fluid were below detection limit.

### Measurement of NO in BAL fluid

NO levels were determined in the BAL fluid. Therefore, the first ml of the lavage fluid was centrifuged and the supernatant was kept at -20°C until analysis. The remaining pellet was resuspended in the recovered lavage fluid and used to determine total and differential cell numbers (see above). Both nitrite, nitrate and S-nitrosothiol levels (NO_x_) in BAL fluid were determined as stable and representative breakdown products of NO [44]. Therefore, samples of 25 μl of BAL fluid were injected into a gas stripping apparatus containing 2 ml of a 1% solution of vanadium (III) chloride in hydrochloric acid at 90°C which was connected to a Sievers 280i NO analyser (Boulder, CO, USA). NO was measured according to the instructions of the manufacturer (Sievers Nitric Oxide Analyzer NO A280, Operational Service Manual, Boulder, CO, USA; Sievers Instruments I 1996) [45]. The sensitivity of the NO analyser is < 10 pmol/ml, with a linearity of 4 orders of magnitude. Calibrations were made according to the manufacturer's instructions with standard solutions of sodium nitrate and sodium nitrate (Sigma), respectively [46].

### Statistical analysis

All data are expressed as mean ± SEM. Differences in eNOS activity, total NO levels in BAL fluid, total and differential cell numbers in BAL fluid, cytokine production by thoracic lymph nodes and IL-5 levels in BAL fluid were tested using the Student's *t*-test (unpaired). Differences between groups after aerosolized methacholine were tested by a general linear model of repeated measurements followed by post-hoc comparison between groups. Data were log transformed before analysis to equalize variances in all groups. Also, the Student's *t*-test (unpaired) was used to determine statistical differences between groups at 25 mg/ml methacholine concentration. All p-values <0.05 were considered to reflect a statistically significant difference.

## Results

### Increased eNOS overexpression and eNOS activity in lungs of eNOS overexpressing mice

**Figure 1:**
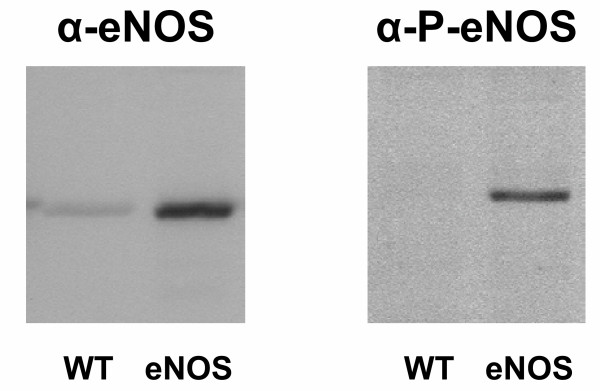
Immunoblotting of lung tissue from WT and eNOS mice. Increased expression of both eNOS (α-eNOS) and phosphorylated eNOS (α-P-eNOS) was found in eNOS mice compared to WT mice. Experiments have been performed at least three times in which similar results were obtained.

The presence of eNOS protein in lung tissue was detected by immunoblotting using two different antibodies. First, using an antibody against human eNOS (α-eNOS), a faint band was observed in lung tissue from WT animals, whereas a much more prominent band could be detected with lung tissue derived from eNOS mice (Fig 1A). Secondly, since the enzymatic activity of eNOS is tightly regulated by different mechanisms, such as the phosphorylation on Ser1179 by the serine/threonine protein kinase Akt [47,48], we also used an antibody against phosphorylated eNOS (α-P-eNOS). As shown in figure 1, endogenous mouse P-eNOS was not detectable in the WT mice, whereas P-eNOS was present in lungs from eNOS mice.

**Figure 2:**
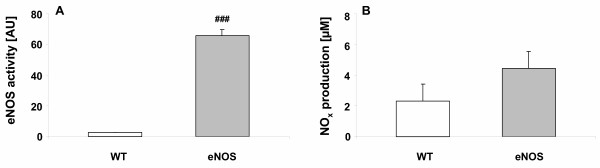
(**A**) eNOS activity in lung homogenates from WT (white bars) and eNOS (hatched bars) mice measured by using the arginine to citrulline conversion assay and (**B**) NO_x _levels measured in BAL fluid of WT mice (white bars) and eNOS mice (hatched bars). Increased eNOS activity was observed in eNOS mice compared to WT mice. NO_x _levels were higher in eNOS mice compared to WT mice. eNOS activity is expressed as mean ± SEM arbitrary units, n = 6. NO_x _data are presented as mean ± SEM, n = 6. ^### ^p < 0.001 compared to WT mice.

We also measured eNOS activity in lungs from WT mice and eNOS overexpressing mice using the L-arginine to L-citrulline conversion assay. The eNOS mice showed a 25-fold increased activity compared to WT mice (Fig 2A). Furthermore, eNOS mice produce more NO in the lungs, since nitrite and nitrate (NO_x_) levels in BAL fluid were increased by 90% in eNOS mice (SAL/eNOS) compared to WT mice (SAL/WT, Fig 2B). From these data we conclude that eNOS is indeed overexpressed in eNOS mice and that eNOS activity is increased in these mice.

### eNOS is localized in endothelium

**Figure 3:**
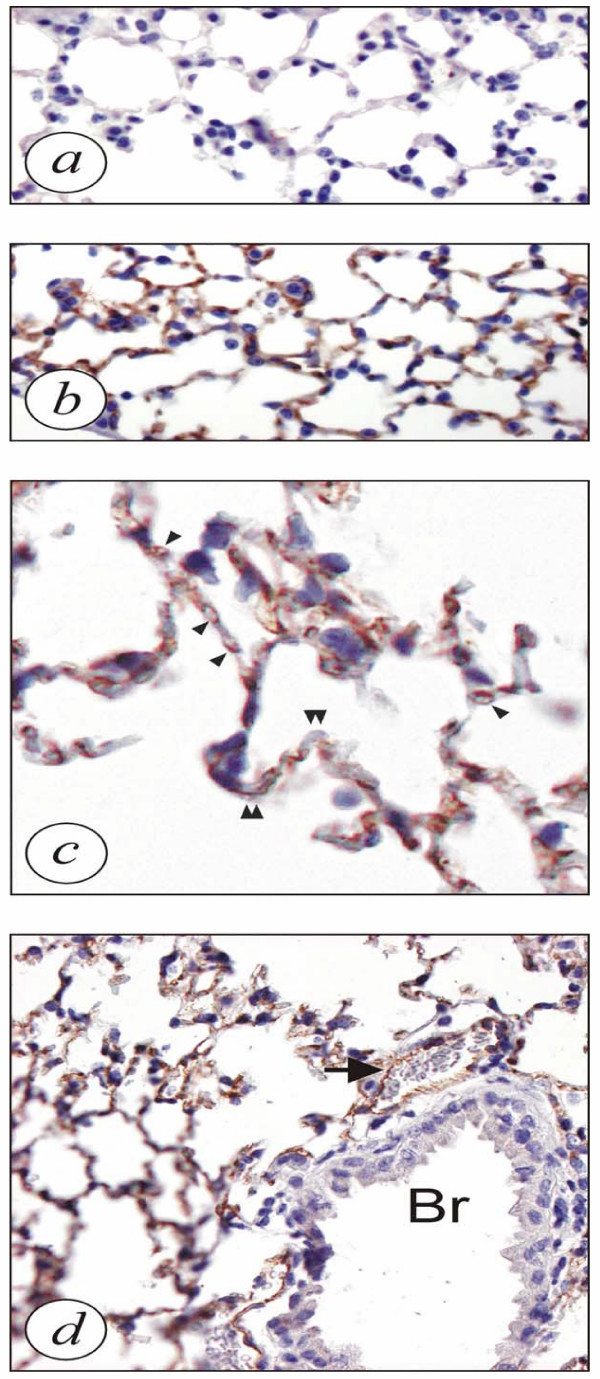
Immunohistochemistry of sections from lung tissue with antibodies directed against human eNOS. **A**) Control mice: Only a faint background staining is present. **B**) eNOS transgenic mice: eNOS is localized in the alveolar septa in lungs. **C**) eNOS transgenic mice: eNOS is present within the septa. Arrowheads point at alveolar capillaries. No signal is seen in the type I cells lining the alveoli (double arrowheads). **D**) eNOS transgenic mice: No signal is seen in the cells from the bronchiole (Br), while the endothelial cells from the small vessel accompanying the bronchiole show staining (arrow). Original magnifications: 100× (A, B, D) and 630× (C). Experiments have been performed at least three times in which similar results were obtained.

The localization of eNOS expressed by the transgenic mice was evaluated by immunohistochemistry studies in lung sections. While the alveolar septa of control mice were only faintly stained, these show a strong signal in transgenic mice (Fig 3A and 3B, respectively). The staining appeared to be present in the interior parts of the alveolar septa, which is clearly visible at a higher magnification (Fig 3C), in which immuno-positive capillaries in the septa can be discriminated while the type I cells that line the alveolar surfaces are not stained. Bronchioles are not stained (Fig 3D).

### eNOS overexpression suppresses the influx of inflammatory cells into the lungs

**Figure 4:**
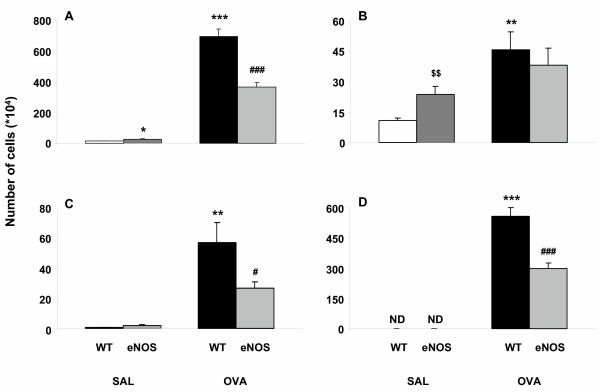
Cell numbers in BAL fluid obtained 24 hours after challenge from SAL/WT mice (white bars), SAL/eNOS mice (hatched bars), OVA/WT mice (black bars) and OVA/eNOS mice (grey bars). **A**) total cell numbers in BAL fluid. Total cell numbers are increased in OVA/WT mice compared to SAL/WT mice. Total cell numbers are decreased by 47% in OVA/eNOS mice compared to OVA/WT mice. **B**) number of alveolar macrophages in BAL fluid. The number of macrophages is increased in SAL/eNOS mice compared to SAL/WT mice. Numbers of macrophages were 4 times increased in OVA/WT mice compared to SAL/WT mice. No difference in macrophages was observed between OVA/eNOS and OVA/WT mice. **C**) number of lymphocytes in BAL fluid. Hardly any lymphocytes could be detected in SAL/WT and SAL/eNOS mice. A markedly increased number of lymphocytes were found in OVA/WT mice compared to SAL/WT mice. A 54% reduction in lymphocytes was found in OVA/eNOS mice compared to OVA/WT mice (p < 0.05). **D**) number of eosinophils in BAL fluid. No eosinophils were present in the BAL fluid of SAL/WT mice and SAL/eNOS mice (ND = not detectable). A dramatic increase in eosinophils was detected in OVA/WT mice compared to SAL/WT mice. Compared to OVA/WT, there was a 46% reduction in the increase in eosinophils in the BAL fluid in OVA/eNOS mice (p < 0.001). Data are presented as mean ± SEM, n = 9. * p < 0.05, ** p < 0.01, ^$$ ^p < 0.01, *** p < 0.001 compared to SAL/WT mice, ^# ^p < 0.05, ^### ^p < 0.001 compared to OVA/WT mice.

To study the effects of eNOS overexpression on the influx of inflammatory cells into the lungs after OVA challenge, total and differential cell numbers in the BAL fluid 24 hours after OVA challenge were determined. Total cell numbers of SAL/WT and SAL/eNOS mice were 13.4 ± 1.2 × 10^4 ^and 25.3 ± 4.7 × 10^4^, respectively (p < 0.05). The number of alveolar macrophages was more than two times higher in SAL/eNOS mice compared to SAL/WT mice (p < 0.01, Fig 4B). Also a small, but not significant (NS, p = 0.09) increase in the number of lymphocytes could be observed in SAL/eNOS mice compared to SAL/WT mice (Fig 4C). Eosinophils (Fig 4D) and neutrophils (data not shown) were not present in BAL fluid of both SAL/WT and SAL/eNOS mice. Aerosol OVA exposure increased the total cell numbers to 695 ± 48 × 10^4 ^in OVA/WT mice and to 366 ± 32 × 10^4 ^in OVA/eNOS mice. Therefore, there was a 47% reduction (p < 0.001) in total cell numbers in OVA/eNOS compared to OVA/WT mice (Fig 4A). The increase in total cell numbers after OVA challenge was mainly due to an increase in the number of eosinophils (80% of total cell population in OVA/WT mice). In OVA/eNOS mice, there was a 46% reduction in the number of eosinophils compared to OVA/WT mice (p < 0.001, Fig 4D). Furthermore, eNOS overexpression reduced the increase in the number of lymphocytes (54%, p < 0.05, Fig 4C) after OVA challenge. However, no difference in the number of macrophages (Fig 4B) and neutrophils (data not shown) could be found in OVA/eNOS mice compared to OVA/WT mice. From these results we conclude that eNOS overexpression reduces the influx of inflammatory cells, predominantly eosinophils, into the lungs after OVA challenge.

### eNOS overexpression does not affect IgE levels in serum

**Figure 5:**
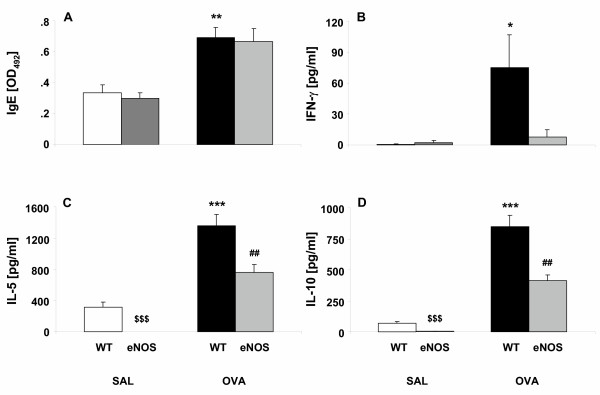
Ovalbumin-specific IgE levels in serum and different production of cytokines by TLN cells after OVA restimulation in vitro in SAL/WT mice (white bars), SAL/eNOS mice (hatched bars), OVA/WT mice (black bars) and OVA/eNOS mice (grey bars). **A**) IgE levels in serum 24 hours after challenge. An increase in IgE was observed after OVA challenge. No difference between eNOS and the respective WT mice was detected. **B**) IFN-γ production by TLN. No IFN-γ was produced by TLN cells obtained from SAL/WT and SAL/eNOS mice. IFN-γ production was markedly increased in OVA/WT mice, but only slightly in OVA/eNOS mice. **C**) IL-5 production by TLN cells. Low levels of IL-5 were found in SAL/WT mice, whereas no IL-5 production was found in SAL/eNOS mice. IL-5 levels were markedly increased in OVA/WT mice compared to SAL/WT mice. IL-5 production was decreased by 44% in OVA/eNOS mice compared to OVA/WT mice (p < 0.01). **D**) IL-10 production by TLN cells. Low levels of IL-10 were found in SAL/WT mice, whereas no IL-10 production was found in SAL/eNOS mice. IL-10 levels were increased in OVA/WT mice compared to SAL/WT mice. IL-10 production was decreased by 51% in OVA/eNOS mice compared to OVA/WT mice (p < 0.01). Data are presented as mean ± SEM, n = 6. * p < 0.05, ** p < 0.01, *** p < 0.001, ^$$$ ^p < 0.001 compared to SAL/WT mice, ^## ^p < 0.01 compared to OVA/WT mice.

eNOS overexpression in SAL challenged mice had no influence on IgE levels in serum (Fig 5A). Antigen challenge induced a significant increase in serum levels of total IgE in both WT mice (p < 0.01) and eNOS mice (p < 0.01), compared to SAL challenged mice (Fig 5A). No difference in serum IgE levels between OVA/WT and OVA/eNOS mice could be observed (Fig 5A). From this we conclude that eNOS overexpression has no effect on IgE levels in serum both after saline and ovalbumin challenge.

### eNOS overexpression suppresses cytokine production by thoracic lymph nodes in vitro

We next compared the cytokine profiles of TLN cells obtained from WT and eNOS mice. eNOS overexpression in SAL challenged mice had no influence on IFN-γ production by TLN cells in vitro (Fig 5B). However, IL-5 production (Fig 5C) was completely absent in TLN cells obtained from SAL/eNOS mice (0.41 ± 0.2 pg/ml) compared to SAL/WT mice (312 ± 70 pg/ml). Furthermore, eNOS overexpression significantly (p < 0.001) reduced in vitro production of IL-10 by TLN (4.74 ± 1.35 pg/ml in SAL/eNOS mice and 68.9 ± 12.1 pg/ml in SAL/WT mice; Fig 5D). OVA challenge highly increased IFN-γ production in WT mice (p < 0.05) compared to SAL/WT, whereas no significant increase in IFN-γ production was observed in cells derived from OVA/eNOS mice compared to SAL/eNOS (Fig 5B). Interestingly, IFN-γ production was decreased by 90% in OVA/eNOS mice compared to OVA/WT mice. After OVA challenge, an increased production of IL-5 and IL-10 (both p < 0.001) by TLN cells in vitro was found in WT mice (Fig 5C + Fig 5D). In OVA/eNOS mice, IL-5 and IL-10 production was decreased by 44% and 51%, respectively, compared to OVA/WT mice (both p < 0.01). From this we conclude that eNOS overexpression attenuates IFN-γ, IL-5 and IL-10 production by TLN cells in vitro after OVA challenge.

### eNOS overexpression attenuates IL-5 levels in BAL fluid

**Figure 6:**
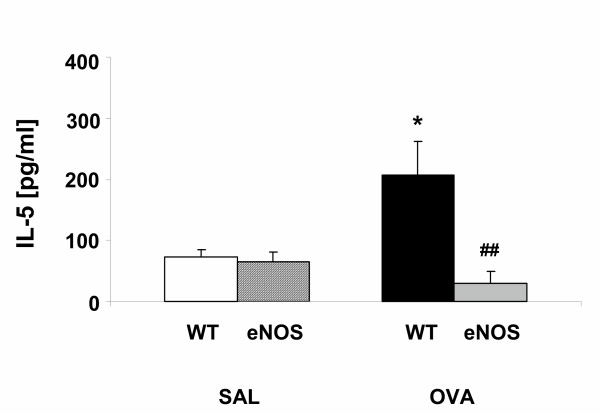
IL-5 levels in BAL fluid in SAL/WT mice (white bars), SAL/eNOS mice (hatched bars), OVA/WT mice (black bars) and OVA/eNOS mice (grey bars). Low levels of IL-5 were found in SAL/WT and SAL/eNOS mice. IL-5 levels were increased in OVA/WT mice compared to SAL/WT mice. IL-5 levels were markedly decreased in OVA/eNOS mice compared to OVA/WT mice. Data are presented as mean ± SEM, n = 6. * p < 0.05 compared to SAL/WT mice, ^## ^p < 0.01 compared to OVA/WT mice.

Next, we measured IL-5 levels in BAL fluid. eNOS overexpression in SAL challenged mice had no influence on IL-5 levels in BAL fluid (Fig 6). IL-5 levels in BAL fluid of OVA/WT mice were significantly increased (p < 0.05) compared to SAL/WT. IL-5 levels in BAL fluid were significantly (p < 0.01) reduced in OVA/eNOS mice compared to OVA/WT mice (Fig 6). We conclude that eNOS overexpression reduces IL-5 levels in BAL fluid after OVA challenge.

### eNOS overexpression prevents the development of airway hyperresponsiveness

**Figure 7:**
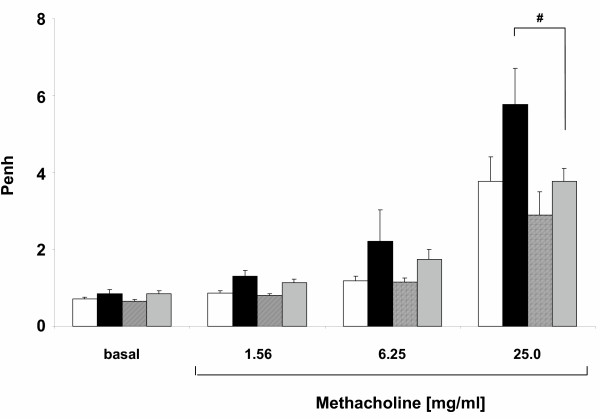
Airway responsiveness (expressed as Penh) to aerosolized methacholine was measured in conscious, unrestrained mice 24 hours after challenge. Increased airway responsiveness to methacholine (p < 0.01) was observed in OVA/WT mice (black bar, n = 7) compared to SAL/WT mice (white bar, n = 9). No effect on airway responsiveness was observed in SAL/eNOS mice (hatched bar, n = 7). Airway hyperresponsiveness was abolished in OVA/eNOS mice (grey bar, n = 11) compared to OVA/WT mice (p < 0.05 at 25 mg/ml methacholine). Data are presented as mean ± SEM. ^# ^p < 0.05 compared to OVA/WT mice.

To investigate the effects of eNOS overexpression on the development of airway hyperresponsiveness, airway responsiveness to methacholine (expressed as Penh values) 24 hours after OVA challenge was measured in unrestrained mice in a Buxco set-up. Basal responsiveness was slightly increased in OVA-challenged mice compared to SAL- challenged mice (0.85 ± 0.11 vs 0.71 ± 0.06 in WT mice and 0.85 ± 0.08 vs 0.65 ± 0.04 in eNOS mice). No difference in airway responsiveness to methacholine between SAL/WT mice and SAL/eNOS mice could be observed (Fig 7). Airway responsiveness to methacholine was significantly (p < 0.01) increased in OVA/WT mice compared to SAL/WT mice (Fig 7). However, no statistically significant difference in airway responsiveness to methacholine could be found between OVA/eNOS mice and SAL/eNOS mice (p = 0.10). In OVA/eNOS mice, there was a 35% reduction in airway responsiveness to 25 mg/ml methacholine compared to OVA/WT mice (p < 0.05, Fig 7). Thus, OVA/eNOS mice are less responsive to high concentrations of methacholine compared to OVA/WT mice.

## Discussion

NO has been implicated in many physiological and pathophysiological processes and it may play a crucial role in airway functioning both during health and disease [19]. In healthy situations, NO derived from cNOS seems to be predominant, since low levels of NO produced by cNOS controls airway smooth muscle tone [23,24,49]. During asthmatic disease however, high levels of NO derived from the iNOS isoform are a major contributor to the inflammatory process seen in asthma [30,34].

Several studies have shown the importance of the different NOS isoforms in the development of asthmatic features, such as increased airway responsiveness, airway inflammation and increased production of several cytokines. Xiong et al [34] again stressed the role of the iNOS isoform in the inflammatory process in asthma. In contrast, De Sanctis et al [50] showed that the iNOS isoform is not important in the development of airway inflammation. Furthermore, Feder et al [51] showed that a selective iNOS inhibitor had no effect on the influx of eosinophils into the lungs of allergen-challenged mice. Moreover, no increase in pulmonary iNOS was found in sensitized and challenged mice. These findings suggest that the iNOS isoform is not the only factor contributing to airway inflammatory processes. In the present study, we have used eNOS overexpressing mice to explore the effects of this NOS isoform on the development of asthmatic features in a mouse model of allergic asthma. Our results show that eNOS plays a crucial role in both airway hyperresponsiveness and airway inflammation.

Overexpression of eNOS in mice was confirmed by several ways. First, immunoblot analysis showed eNOS expression in the lungs of overexpressing mice, however, this protein was hardly found in wild type mice. Using the L-arginine to L-citrulline conversion assay, it was demonstrated that eNOS activity was significantly increased in the lungs of overexpressing mice. NO_x _levels were more than doubled in the bronchoalveolar lavage fluid of overexpressing mice and immunohistochemistry indicated an increased expression of eNOS in the lungs. These results indicate that eNOS-derived NO has functional properties in the lungs of these mice [52].

NO itself has a very short half-life and reacts rapidly with many different molecules in a biological environment. S-nitrosothiols are important molecules signaling NO bioactivity in the airways. Low levels of S-nitrosothiols are associated with severe asthma [53], and recently, Que et al. [21] showed that allergic mice that cannot metabolize S-nitrosothiols are completely protected from airway hyperresponsiveness. Moreover, S-nitrosothiols repressed the action of inhibitory κB kinase, providing a mechanism for the anti-inflammatory properties of NO [54]. We can speculate that overexpression of eNOS in the airways leads to higher concentrations of NO and the consequent higher levels of S-nitrosothiols in the lungs.

Airway inflammation is the key factor in the pathogenesis of asthmatic disease [55]. Besides other inflammatory cells, the eosinophil is thought to be one of the major effector cells in asthma. In the present study we found that eNOS overexpression reduced the influx of eosinophils into the lungs after ovalbumin challenge by 46%. A direct effect of NO on lung eosinophilic influx is doubtful. Although chemical inhibition of NO activity has been shown to suppress pulmonary eosinophilic inflammation in mice [51,56], in NOS1, 2 and 3 KO mice no difference in the number of lung eosinophils could be observed [50]. Eosinophil mobilisation and trafficking are largely promoted by the Th2 cytokine IL-5 [57]. Ablation of the effects of IL-5 has been accomplished with blocking anti-IL-5 antibody, which was accompanied by a reduction in allergen-induced eosinophilia [58-60]. We found that IL-5 production by TLN cells in vitro is reduced by 44% in OVA/eNOS mice. Furthermore, IL-5 was almost completely absent in the BAL fluid of these mice. The attenuated IL-5 production might account for the reduced presence of eosinophils in the lungs. Interestingly, IL-5 production by TLN cells was completely absent in SAL/eNOS mice. Therefore, eNOS overexpression might, via inhibition of IL-5 production, attenuate the maturation of eosinophils in the bone marrow [51,61].

In the present study, we also found reduced levels of lymphocytes in the BAL fluid of OVA/eNOS mice. Although we did not measure numbers of circulating white blood cells, it has been reported that NO inhibits leukocyte adhesion and migration through the endothelial cell layer [62]. Since immunohistochemical data showed overexpression of eNOS predominantly in the endothelium, an NO-induced decrease in leukocyte adhesion might, at least partly, offer an explanation for the decreased airway inflammation in this mouse strain. Moreover, NO attenuates T cell proliferation [63,64] and, at high levels, NO can induce T cell apoptosis through S-nitrosylation of different target proteins [65,66].

Airway hyperresponsiveness is a well-established characteristic of allergic asthma and is believed to be the result of airway inflammation as well as epithelial damage [23,67]. Interestingly, several studies have shown that eNOS KO mice are hyperresponsive to inhaled bronchoconstrictor agents like methacholine [49,50]. We show in the present study that the development of airway hyperresponsiveness was completely prevented in OVA/eNOS mice at a high methacholine concentration. It is not unlikely that this is due to the fact that only at high concentration of a bronchoconstricting agent, high levels of NO are necessary to counteract this constriction. This idea is supported by the observation that eNOS overexpression has no effect on basal responsiveness to methacholine.

A disbalance between Th2 and Th1 lymphocytes seems to be correlated with the development of atopic diseases [8,17,68]. mRNA expression data in BAL cells from atopic asthmatics showed a predominant Th2 cell like pattern [8,10], with the consequent elevation of IL-4 and IL-5 [69]. Furthermore, Th1 cells are thought to antagonize Th2 cell functions [7]. Previously, a role for iNOS in the Th1/Th2 balance was proposed, since in iNOS KO mice a suppression of allergic inflammation was found, which was accompanied by an increased IFN-γ production by T cells [34]. However, other studies showed that antigen-specific Th1 cells do not protect or prevent Th2-mediated allergic diseases, but rather may cause acute lung pathology [14,15,70]. Furthermore, IFN-γ levels are elevated in serum [12] and BAL fluid [11,71] of patients with asthma. Interestingly, treatment with antibodies to IFN-γ completely abolished airway hyperresponsiveness, but had no effect on airway eosinophilia [59]. In contrast, other studies have shown that anti-IL-5 blocks eosinophilic influx into the lungs, although hardly any effect on airway hyperresponsiveness could be observed [59,72]. In the present study, we found attenuated levels in OVA/eNOS mice of both the Th1 cytokine IFN-γ and the Th2 cytokines IL-5 and IL-10 compared to OVA/WT mice. Therefore, NO derived from eNOS might be involved in the attenuated production of both Th1 and Th2 cytokines, resulting in diminished airway inflammation and, although not causally related [21], this might reduce the development of airway hyperresponsiveness. Indeed, NO inhibits the secretion of IFN-γ by Th1 cells [32,73,74] and IL-5 and IL-10 by Th2 cells [75] by affecting several signaling molecules and transcription factors in T cells (recently reviewed in [76]).

The role of IL-10 in asthma remains controversial. Some studies found a higher IL-10 expression in subjects with asthma than in control subjects [77], whereas others found lower IL-10 levels [78]. Furthermore, although some studies show a close relationship between IL-10 and iNOS levels [79], no data exists showing a role for IL-10 and eNOS expression. In the present study, we demonstrated a significantly lower IL-10 production by TLN cells in SAL/eNOS mice and OVA/eNOS mice compared to their respective controls. The attenuated IL-10 production in eNOS mice might contribute to the reduced development of asthmatic features in this asthma model.

Atopic individuals can be recognized by the presence of allergen-specific IgE in their serum and by elevations of the total serum IgE [6]. Indeed, we found elevated levels of total IgE in serum after ovalbumin challenge, but we could not observe any differences between OVA/WT and OVA/eNOS mice. These results confirm the finding by De Sanctis et al [50], who observed no difference between IgE levels in several NOS isoform KO mice after challenge with ovalbumin compared to WT mice.

## Conclusion

We have shown that overexpression of the eNOS gene prevents the development of airway hyperresponsiveness, airway inflammation and the production of Th1 and Th2 cytokines in a mouse model of allergic asthma. Although it has been assumed for many years that iNOS has a primary role in inflammatory diseases like asthma [30,32,80], recent studies suggest an important role for the cNOS isoforms in controlling asthmatic disease [22,50,51,81]. Interestingly, eNOS gene polymorphisms have been associated with atopic asthma [35,38], with lower NO concentrations leading to persistent airway inflammation [37]. The eNOS gene therefore seems a promising new target for new insights and new opportunities for improvements in therapy against asthmatic disease.

## Competing interests

The author(s) declare that they have no competing interests.

## Authors' contributions

RTB participated in the design and coordination of the study and drafted the manuscript. RDC and RVH generated the eNOS transgenic mice and performed eNOS activity and Western blot analysis. VV, TLM and IVA carried out the all lung experiments. FDC, FN and GF helped to draft the manuscript and participated in the coordination and analysis of the study. All authors read and approved the final manuscript.
